# Computed Tomography and Magnetic Resonance Imaging in Liver Iron Overload: From Precise Quantification to Prognosis Assessment

**DOI:** 10.3390/biomedicines12112456

**Published:** 2024-10-25

**Authors:** Xinrui Zhou, Xinyuan Jia, Yidi Chen, Bin Song

**Affiliations:** 1 Department of Radiology, West China Hospital, Sichuan University, Chengdu 610041, China; zhouxstar@163.com (X.Z.); jiaxinyuan54@163.com (X.J.); 2 Functional and Molecular Imaging Key Laboratory of Sichuan Province, West China Hospital, Sichuan University, Chengdu 610041, China; 3 Department of Radiology, Sanya People’s Hospital, Sanya 572000, China

**Keywords:** liver iron overload, computed tomography, magnetic resonance imaging, precise quantification, prognosis assessment

## Abstract

Liver iron overload is associated with conditions such as hereditary hemochromatosis, thalassemia major, and chronic liver diseases. The liver-related outcomes, patient outcomes, and treatment recommendations of these patients differ depending on the cause and extent of iron overload. Accurate quantification of the liver iron concentration (LIC) is critical for effective patient management. This review focuses on the application of computed tomography (CT) and magnetic resonance imaging (MRI) for the precise quantification and prognostic assessment of liver iron overload. In recent years, the use of dual-energy CT and the emergence of MRI-based sequences (such as UTE, QSM, Dixon, and CSE technologies) have significantly increased the potential for noninvasive liver iron quantification. However, the establishment of internationally standardized imaging parameters, postprocessing procedures, and reporting protocols is urgently needed for better management of patients with liver iron overload.

## 1. Introduction

Iron overload can generally be categorized into two main types: primary and secondary [1,2,3]. Primary iron overload is associated with mutations in the *HFE* gene [1,4,5], leading to hereditary hemochromatosis (HH), an autosomal recessive genetic disorder. Secondary iron overload includes conditions requiring long-term transfusion therapy, such as thalassemia and iron-deficiency anemia, as well as chronic liver diseases like non-alcoholic fatty liver disease (NAFLD) and alcoholic liver disease (ALD), which involve distinct underlying mechanisms [6,7,8]. Physiologically, iron is stored within hepatocytes as bound ferritin. Excessive iron, however, remains free and can deposit in various organs, including the brain, liver [9], heart, pancreas, and endocrine glands. These deposits can trigger several diseases, such as neurological disorders, liver diseases, cardiomyopathy, and type II diabetes [10,11]. Treatment methods include phlebotomy therapy and chelation therapy [12,13], both aimed at reducing the total iron concentration.

The liver, as the main organ for iron storage, is often the first to be impacted. Iron deposition in the liver can damage liver cells, leading to fibrosis and cirrhosis, and can exacerbate pre-existing liver damage in patients with chronic liver disease. Furthermore, iron overload also serves as an independent risk factor for the development of hepatocellular carcinoma (HCC) in patients with end-stage liver disease of various etiologies [14]. Moreover, excessive iron accumulation in the heart induces oxidative stress, damaging cardiomyocytes and the cardiac conduction system, which can progress from initially asymptomatic stages to arrhythmias and ultimately lead to heart failure [15]. In hemophilic arthropathy [16], although iron overload is not the underlying cause, repeated joint hemorrhages result in localized iron deposition in joint cartilage, bone, and surrounding soft tissues. This deposition triggers chronic inflammation, joint destruction, and functional impairment, severely affecting joint mobility. Clinically, liver iron concentration (LIC) is a crucial indicator for the diagnosis and treatment of iron overload. It has been reported to correlate strongly and linearly with total body iron levels [1]. Accurate quantification of LIC is therefore essential for diagnosis, grading severity, guiding iron-reducing therapies, and monitoring treatment response.

**Figure 1:**
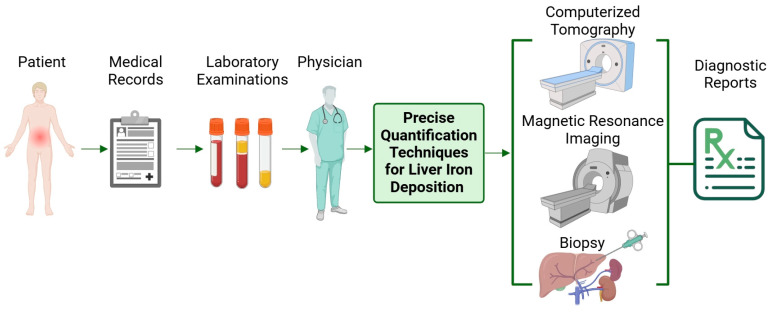
The workflow of clinical practice for precise quantification of liver iron deposition. The level of serum ferritin cannot accurately assess the degree of iron deposition in the liver. Computerized tomography, magnetic resonance imaging, and tissue biopsy provide recognized and effective techniques for the quantitative detection of iron deposition in the liver.

Currently, many methods are available to quantify LIC, ranging from laboratory tests, such as serum ferritin (SF) and transferrin saturation (TSAT), to noninvasive imaging techniques like superconducting quantum interference device (SQUID) [17], dual-energy CT (DECT) [18], MRI technologies [19], and invasive liver biopsy (the current gold standard). However, SF and TSAT are prone to be influenced by alcoholism, inflammation, several chronic liver diseases and tumors [20]. Mechanisms leading to elevated SF and TSAT in conditions other than HH differ from those causing HH-induced iron overload. Multiple factors, including stimulation by inflammatory cytokines, hepatocyte damage, and alterations in hepcidin expression, collectively contribute to the increased levels of SF and TSAT. Furthermore, these elevations may occasionally be an acute reactive response rather than a real increase in body iron concentration [21]. Additionally, serum biochemical tests cannot reflect iron deposition in each organ. Liver biopsy [19], while straightforward, is invasive and subject to sampling errors, making it difficult to use it longitudinally and widely. In addition, many patients with iron overload also suffer from thrombocytopenia, which is a contraindication for liver biopsy. The use of these methods in clinical practice in order of priority can be featured in Figure 1.

In recent years, noninvasive imaging techniques have been increasingly recognized for their potential in iron quantification. Table S1 in the Supplementary Materials shows the cutoff criteria for classifying the severity of hepatic iron overload via several common methods.

This review aims to summarize the effectiveness of noninvasive imaging technologies in quantifying LIC, with a particular focus on DECT and MRI, from precise quantification to prognosis assessment.

## 2. Precise Imaging Quantification Techniques for Liver Iron Deposition

### 2.1. Dual-Energy CT

**Figure 2:**
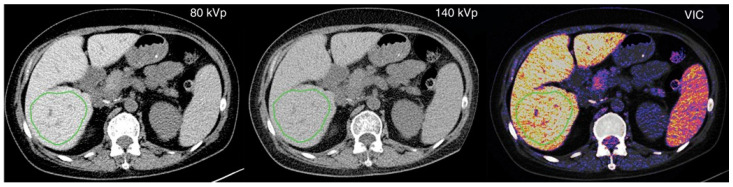
CT measurement in 54-year-old woman with myelodysplastic syndrome. Mean attenuation in liver was measured by placing largest possible freehand regions of interest at segments V and VI of right hepatic lobe. CT measurements were 117.8, 65.3, and 54.1 HU at 80 kVp imaging, 140 kVp imaging, and VICvirtual iron content imaging, respectively. Reproduced with permission from Luo XF et al. [18].

With the advancement of CT technology, representative techniques include single-source rapid kVp switching spectral CT and dual-source dual-energy computed tomography (DSDE CT). DSDE CT, as an emerging technique for evaluating liver iron content, is based on the principle of differential X-ray absorption by different substances and the quantification of tissue differences in the energy domain, enabling the assessment of liver iron concentration. DSDE CT utilizes a dual-energy (80/140 kVp) scanning mode for liver imaging [22]. The main quantitative measurement methods include the dual-energy CT difference method (ΔH method) and the three-material decomposition algorithm. The utilization of DECT can be demonstrated in Figure 2.

The ΔH method calculates the difference in liver CT values between 80 kVp and 140 kVp images to estimate the substance composition and content via the obtained ΔH value. Joe et al. [23] reported that ΔH is significantly correlated with the degree of hepatic iron accumulation, but this method has low specificity in assessing low-concentration liver iron deposition due to the influence of hepatic fat variation.

On the other hand, the three-material decomposition algorithm uses iron-specific slope reconstruction to generate a VIC map of the liver, reducing interference from fat variation. Luo et al.’s [18] findings revealed a strong association between liver VIC values and serum ferritin levels. Hepatic VIC values have emerged as a potential index for accurately assessing liver iron accumulation and exhibit diagnostic accuracy comparable to that of MRI.

The application of DECT imaging technology, with its ability to perform material separation, plays a crucial role in achieving precise quantification of the liver iron LIC and has significant implications in the assessment of liver iron overload [24]. However, DECT has not gained wide acceptance as a routine standard for clinical liver iron quantification because of its insufficient quantitative accuracy and the potential risks associated with ionizing radiation.

Diffuse liver disease is often associated with fat deposition in addition to increased iron content. When hepatic steatosis occurs, fat could be a compounding factor in the estimation of hepatic iron [18]. Fat results in a decrease in attenuation of the hepatic parenchyma, confounding the effect of iron. Therefore, routine computed tomography shows a lack of sensitivity for iron detection and underestimation of LIC in the presence of fat. In contrast to routine CT, an animal study demonstrated that DECT can successfully differentiate coexisting hepatic iron and fat [25]. By employing an iron specific, three-material decomposition algorithm, DECT enables the quantification of LIC regardless of the fat content. This is achieved by generating VIC images that effectively eliminate the confounding effect of fat. The efficacy of VIC imaging in eliminating the influence of fat has been previously confirmed in an ex vivo phantom study [26].

### 2.2. MRI

MRI offers a noninvasive alternative for the assessment of LIC. Owing to the paramagnetic properties of iron, the transverse relaxation of magnetization from protons in water is accelerated, resulting in a concentration-dependent decrease in signal intensity, commonly referred to as “darkening” on T2- and T2*-weighted images. The decay of signal intensity in spin-echo MRI and gradient–echo MRI is characterized by the time constants T2 and T2*, respectively, both measured in milliseconds. T2 is a parameter that characterizes the intrinsic decay of transverse magnetization due to spin-spin interactions. On the other hand, T2* incorporates the influence of local magnetic field inhomogeneities in addition to the intrinsic decay: 1/T2* = 1/T2 + 1/T2′, where T2′ represents the supplementary contribution from field inhomogeneities. These parameters, T2 and T2*, can also be expressed as relaxivity rates, denoted as R2 = 1/T2, R2* = 1/T2*, and R2* = R2 + R2′. For example, a T2* value of 5 msec corresponds to an R2* value of 200 s^−1^ (i.e., R2* = 1/0.005 s). Transverse relaxometry methods, which involve acquiring signal measurements at multiple echo times (TEs) to estimate the decay time are commonly employed for quantifying the liver iron concentration. In the context of LIC quantification, the preferred parameter is the rate of signal decay, namely, R2 (1/T2) or R2* (1/T2*), both of which are expressed in units of s^−1^, as they monotonically increase with increasing LIC [27,28]. The current methods commonly used for quantifying liver iron overload include “signal–intensity ratio” (SIR) methods, R2-based relaxometry, and R2*-based relaxometry. Although liver iron quantification techniques can be performed at both 1.5 T and 3.0 T magnetic field strengths, notably, the rate of signal decay is higher at 3.0 T than at 1.5 T. This increased rate of signal decay at 3.0 T enhances the sensitivity to detect the presence of iron, especially at lower iron concentrations [29]. However, in cases of severe iron overload, the dynamic range may become restricted at 3.0 T, making 1.5 T imaging preferable in such situations [28]. Apart from 1.5 T and 3.0 T, the emergence of 7.0 T MRI offers potential significance for detecting iron overload. The 7.0 T MRI system not only offers a higher signal-to-noise ratio but also provides enhanced intrinsic contrast between tissue susceptibilities, enabling ultrahigh-spatial-resolution susceptibility-weighted imaging (SWI). This ultrahigh-resolution SWI allows for clearer visualization of fine structures containing paramagnetic substances such as iron and facilitates the earlier detection of subtle changes, such as liver iron overload.

#### 2.2.1. Liver-to-Muscle Signal Intensity Ratio (SIR)

SIR methods rely on the observation of a decrease in signal intensity caused by T2* shortening when liver iron overload is present, whereas the signal intensity of a reference tissue (typically the paraspinal muscles) is assumed to be unaffected by iron content [19,30]. Normal liver parenchyma, which is free from iron overload, should consistently exhibit higher signal intensity than the paraspinal muscles. Hence, a hypointense liver in relation to the paraspinal muscles indicates the presence of iron overload. This assessment is semiquantitative and involves measuring the liver-to-muscle signal intensity ratio [31]. In cases of severe iron overload, the liver exhibits lower signal intensity than the paraspinal muscles do, even when sequences with limited sensitivity to iron overload, such as T1-weighted and proton-density-weighted sequences, are used. Moderate iron overload is characterized by lower liver signal intensity relative to the paraspinal muscles, and sequences with moderate sensitivity to iron overload, such as T2*-weighted sequences with intermediate TEs, are used. Mild iron overload is indicated by lower liver signal intensity than paraspinal muscle signal intensity when sequences that are highly sensitive to iron overload are used, such as heavily T2*-weighted sequences with long TEs [32]. The LIC value can be determined via free software such as MRQuantif 2024.10.01 (available at https://imagemed.univ-rennes1.fr/ (accessed on 15 September 2024)) or via the Spanish Society of Abdominal Imaging (SEDIA) website https://www.sedia.es/calculador-de-hierro-hepatico-en-rm/ (accessed on 15 September 2024) [30].

The main advantage of SIR methods is the simplicity of implementation across multiple vendors and platforms [28]. The SIR methods offer a practical alternative in situations where high-quality phased-array coils or relaxometry methods are not accessible, also offering a straightforward approach for quantifying liver iron overload, requiring minimal postprocessing. The developers of the technique provide the necessary acquisition parameters and postprocessing calculations without charge. Moreover, when performed at 3.0 T, this method demonstrates enhanced sensitivity in detecting even milder liver iron overload [19,31,33]. However, the dynamic range of valid LIC quantification is limited to approximately 350 μmol/g (19.5 mg/g). This technique is not accurate for severe iron overload exceeding 350 μmol/g and tends to overestimate mild and moderate liver iron overload [19,34]. Additionally, the technique is dependent on the assumption that the reference tissue (muscle) is normal, whereas paraspinal muscles are commonly presumed to remain unaffected. Issues such as muscle atrophy and fat infiltration, especially prevalent in elderly patients, can introduce complicating factors affecting the T1 and T2* measurements of the muscles. Moreover, the existence of spatial signal inhomogeneity caused by B0 or B1 inhomogeneity effects at a 3.0 T magnetic field strength could present an additional limitation to the precision and reliability of the technique [19,33].

#### 2.2.2. T2 and R2 Relaxometry

In the context of iron overload, two theories have been proposed to account for the observed T2 shortening. According to the first theory, the relaxation rate of protons is locally influenced by the exchange of protons between bulk water and exchangeable protons located on the surface of the ferritin core [35]. The second theory proposes that magnetic field inhomogeneity induced by hemosiderin clusters leads to a relaxation mechanism based on proton diffusion [36]. These mechanisms likely contribute to the decrease in T2 and the increase in R2 when iron is present. The technique known as R2 relaxometry, validated by St Pierre et al. [37] and approved by the U.S. Food and Drug Administration (FDA), is commercially available as FerriScan (Resonance Health) [38]. At the same time, R2 values are based on the FerriScan™ technique.

**Figure 3:**
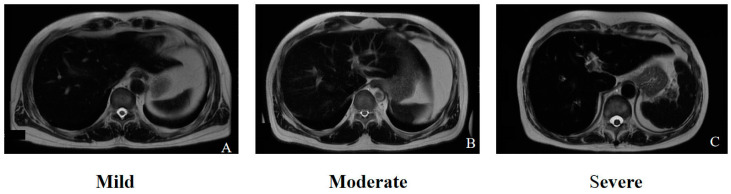
The T2-weighted sequences in (**A**–**C**) correspond to the respective severity levels (mild, moderate, and severe) of liver iron overload in the liver parenchyma.

R2-based LIC quantification is well established as a noninvasive MRI-based reference standard that is widely used in clinical practice and clinical trials [39] because of its good reproducibility [40] and regulatory approval in many countries [41,42]. R2-based relaxometry is effective for assessing the severity of a broad range of iron overloads. R2 mapping shows a strong correlation with the LIC at 1.5 T (r = 0.98) across a wide range of LICs from 0.3 to 42.7 mg/g [37]. However, it is suboptimal for quantifying liver iron concentrations above approximately 40 mg/g (700 μmol/g) [28]. Other limitations of the technique include a relatively lengthy acquisition time (10–20 min), limited spatial coverage of the liver, susceptibility to motion artifacts during free-breathing acquisitions, and exclusive operation at a 1.5 T field strength. Consequently, the widespread implementation of R2-based LIC quantification in various clinical settings remains somewhat limited [27]. Figure 3 elucidates the heterogeneous presentations of liver iron overload on T2 imaging.

#### 2.2.3. T2* and R2* Relaxometry

Iron overload in the liver parenchyma results in a reduction in T2* and an increase in R2* values. This phenomenon arises from a blend of T2 (R2) relaxation impacts and the microscopic inhomogeneities induced in the main magnetic field (B0) by the superparamagnetic characteristics of hemosiderin clusters [43]. In multiple studies, R2* relaxometry, which employs three-dimensional, multiecho, spoiled gradient-recalled echo acquisitions, has gained recognition as a clinically feasible alternative to R2 relaxometry [27]. Advancements in parallel imaging, high-performance gradient hardware, and phased-array receiver coil technology have enabled comprehensive liver coverage within a single short breath hold [43]. The LIC via R2* relaxometry can be determined via various software tools. In a retrospective study, three different programs—FuncTool, CMRtools/Thalassemia Tools, and Quanta Hematology—were used to assess R2* LIC values. FuncTool computes T2*/R2* values (mean and SD) on the basis of a manually selected ROI in the liver, typically analyzing a multiecho gradient sequence with 16 echoes. The R2* values from CMRtools/Thalassemia Tools were converted to LIC values via the equation developed by Garbowski et al. [44]. In Quanta Hematology, LIC values are calculated within a user-defined ROI, which can be drawn in freehand, elliptical, or rectangular shapes [45].

**Figure 4:**
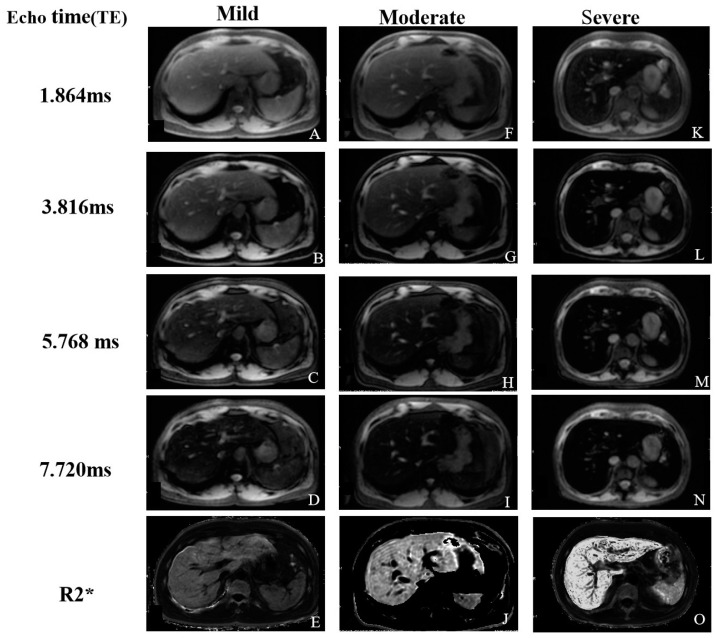
In three patients with different degrees of liver iron overload (mild, moderate, and severe), the provided images consisted of axial gradient-echo (GRE) MR images acquired at 3.0 T, utilizing multiple echo times (TEs) (1.864, 3.816, 5.768, and 7.720 ms). These images are accompanied by corresponding R2* maps. (**A**–**E**): Mild iron overload in a 45-year-old man, using precise quantitative MRI techniques, T2* = 3.018 ms, R2* = 274.1 Hz. (**F**–**J**): Moderate iron overload in a 47-year-old man, T2* = 3.012 ms, R2* = 283.9 Hz. (**K**–**O**): Severe iron overload in a 53-year-old woman, T2* = 1.059 ms, R2* = 721.6 Hz. Iron overload in the liver parenchyma leads to a decrease in T2* and an increase in R2* values. As the degree of liver iron overload increases, there is a corresponding intensification of T2* reduction and R2* elevation in three cases.

Importantly, R2* relaxometry can be performed at both 1.5 T and 3.0 T, maximizing access to available MRI systems. R2* is linearly correlated with the liver iron content determined by biopsy, indicating that R2* relaxometry is a dependable method for noninvasively quantifying liver iron overload [43,46,47,48]. Unfortunately, liver R2* mapping is susceptible to various confounding factors that introduce errors and variability in liver iron quantification. One significant confounder is hepatic steatosis, characterized by excessive triglyceride deposition in the liver, which can contribute to apparent MRI signal decay and introduce bias in R2* measurements if not appropriately accounted for [49]. Additionally, fitting R2* values from magnitude spoiled gradient-recalled echo signals may be prone to noise-related bias if the effects of the noise floor are not adequately addressed during the fitting procedure [50]. Moreover, R2*-based relaxometry may not be well suited for the quantification of LICs greater than approximately 40 mg/g (700 μmol/g) at 1.5 T or approximately 26 mg/g (466 μmol/g) at 3.0 T [28]. The different degrees of liver iron overload (mild, moderate, and severe) are shown in Figure 4.

MRI does not directly measure liver iron levels but rather provides an indirect estimation by assessing the concentration-dependent impact of iron on proton signal decay rates. Consequently, any factor, such as hepatic steatosis, that influences the apparent rate of signal decay can potentially hinder the accurate, precise, and reproducible quantification of tissue iron via MRI [49]. The coexistence of fat and water in the tissue results in signal intensity fluctuations due to interference during gradient-echo acquisition. The presence of fat can introduce bias in LIC estimates derived from the R2* or SIR methods, complicating the apparent relaxation of the fat, as fat decays more slowly than lean liver tissue does [51]. In spin-echo acquisitions, the presence of fat can also introduce challenges in interpreting tissue relaxation behavior, owing to its slower decay rate than that of lean liver tissue. Fibrosis may also confound iron estimation, as it decreases R2 and R2* relaxation rates [31,52], but the magnitude of this effect is much smaller than the range of values observed in iron overload. This may explain why some studies do not find a significant impact of fibrosis on R2* measurements [46,53].

The proton density fat fraction (PDFF) has emerged as the leading noninvasive quantitative imaging biomarker (QIB) for hepatic steatosis [54,55]. It is an objective measure of the tissue triglyceride concentration and is calculated as the ratio of MR imaging-visible triglyceride protons to the sum of triglyceride and water protons. While MR spectroscopy can measure PDFF in small liver tissue volumes, it poses technical challenges such as biased volume selection in livers with nonuniform fat distribution and difficulty in colocalizing measurements over time. To overcome these challenges, chemical shift-encoded MR imaging (CSE-MRI) methods have been developed to automatically map hepatic PDFF values pixel by pixel across the entire liver [56]. These methods, which are commercially available on many MRI systems, are widely used for detecting, grading, and monitoring liver fat [57]. Addressing confounding factors such as T1 variations [58], noise-related bias [58,59], fat spectral complexity [60,61,62], eddy currents [63,64], concomitant gradients [65], and R2* signal decay ensures unbiased and precise PDFF measurements. Confounder-corrected CSE-MRI techniques demonstrate high reproducibility across protocols, vendors, and field strengths [56,66]. A prospective single-center study by Hernando et al. [67] demonstrated that the confounder-corrected CSE-MRI method successfully validated R2* as an accurate and reproducible biomarker for quantifying the liver iron concentration.

In addition to the mentioned MRI sequences, susceptibility-weighted imaging (SWI) and ultrashort-TE sequences (UTEs) have been investigated in certain studies for their potential in the quantitative assessment of hepatic iron deposition. However, further research data are still needed to support their practical value and clinical applicability.

#### 2.2.4. Susceptibility-Weighted Imaging (SWI) and Quantitative Susceptibility Mapping (QSM)

**Figure 5:**
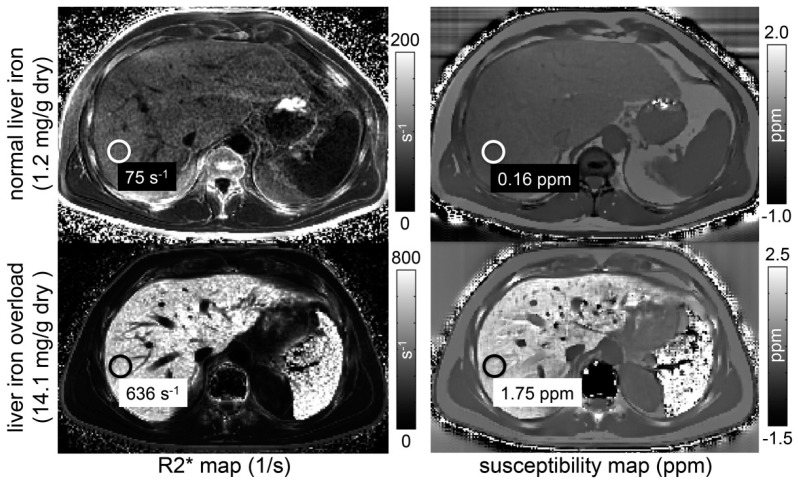
Both MRI−based R2* mapping and quantitative susceptibility mapping are sensitive to the presence of iron in the liver. The figure shows R2* maps (**left**) and susceptibility maps (**right**) estimated from a subject with normal liver iron (**top**) and with liver iron overload (**bottom**). Super−conducting quantum interference device–biomagnetic liver susceptometry estimates of liver iron concentration are reported. The susceptibility measurements reflect the change in liver susceptibility due to the presence of iron. For the subject with liver iron overload, notice the increase in R2* and susceptibility in the spleen, suggesting the presence of iron overload in that organ as well. Re−produced with permission from Sharma SD et al. [69].

SWI, which is based on the T2*-weighted gradient echo sequence, provides enhanced image contrast by exploiting the magnetic susceptibility differences between tissues. It simultaneously generates phase images and magnitude images [68]. The phase images obtained from SWI can be used to quantitatively analyze phase shift changes caused by the magnetic susceptibility effects of substances, indirectly reflecting their relative content. The phase values from SWI can noninvasively and quantitatively measure the degree of liver iron deposition in patients and serve as a precursor for quantitative susceptibility mapping (QSM), which is an emerging MRI technique for quantitatively assessing tissue magnetization properties [69]. QSM enables effective quantitative analysis of iron content, calcifications, and other tissue characteristics. It combines techniques similar to water-fat separation and removes the influence of the main magnetic field on the phase during reconstruction, allowing for direct quantitative measurement of liver magnetization values. Studies have shown significant correlations between QSM measurements and LIC values measured by SQUID and R2* values, with correlation coefficients of 0.88 and 0.94, respectively. Figure 5 displays R2* and susceptibility maps for one participant with typical LIC levels and another participant exhibiting moderate liver iron overload, with SQUID-BLS as the reference standard [68,70]. Compared with gradient echo sequences, QSM may be the most direct and sensitive MRI technique for detecting iron deposition, particularly in cases of mild liver iron deposition. The technique’s postprocessing is based on a single breath hold, minimizing motion artifacts, and some algorithms can correct for the presence of fat. However, the QSM is utilized primarily in research and is not yet standardized or commercially available from major MRI manufacturers. The precise correlation between the QSM values and the liver iron content remains uncertain. Future studies are needed to assess its accuracy, reproducibility, and potential as a valuable technique for quantifying liver iron.

#### 2.2.5. Ultrashort TE Sequences

Gradient-echo acquisitions, either two- or three-dimensional, with multiple TEs within a single repetition, allow for rapid mapping of R2* across the entire liver in just one breath hold [71]. The selection of appropriate TEs is crucial for accurate R2* quantification, especially in the presence of a high LIC requiring short TEs or in the presence of liver fat causing non-monoexponential signal decay. For a high LIC, a short first TE (<1 ms) is necessary, and even shorter TEs may be required at 3.0 T [28]. Additionally, using short TE spacing (<1 ms) is essential to capture rapid signal decay in the presence of high LIC and to correct for the influence of fat. Typically, a total of 6 to 12 echoes is recommended to balance the need for adequate R2* decay sampling while minimizing the increase in repetitions and acquisition time associated with a greater number of echoes.

Ultrashort TE sequences (UTE) with extremely low TE values, as short as 0.1 msec, can be achieved and enable the quantification of significant iron overload, even at 3.0 T. In contrast, the shortest TE achievable with current gradient echo sequences typically falls within the range of 0.8 msec [72]. Initial findings indicate that ultrashort TE sequences have the potential to serve as a feasible substitute for, or alternative to, conventional GRE sequences for accurate R2* estimation, which is applicable to both low and high levels of iron overload [73].

Given the details discussed previously, DECT and various MRI approaches present their own benefits and constraints when assessing liver iron overload. The Table S2 in the Supplementary Materials summarizes these aspects.

## 3. Predictive Value of Liver Iron Imaging Quantification Techniques for Disease Progression, Prognosis, and Treatment Response Assessment

Iron overload disorders include a variety of conditions that cause systemic iron accumulation and organ damage. Primary iron overload is predominantly caused by hereditary hemochromatosis. Secondary iron overload, on the other hand, arises from various hematologic disorders, medical interventions, or chronic liver diseases. Among the more common hematologic disorders that contribute to secondary iron overload are thalassemia syndrome, myelodysplastic syndrome, myelofibrosis, sideroblastic anemias, sickle cell disease, and pyruvate kinase deficiency [3].

The EASL clinical practice guidelines [74] indicate that hemochromatosis is characterized by elevated transferrin saturation and progressive iron accumulation, primarily in the liver. Without treatment, patients face the potential for significant tissue harm from iron toxicity. Regular venesection, involving an initial series of intensive weekly phlebotomies to deplete iron reserves followed by maintenance phlebotomies to prevent iron re-accumulation, can effectively decrease morbidity and mortality risks. Early diagnosis and timely treatment through phlebotomy can help prevent complications such as cirrhosis and hepatocellular carcinoma. A retrospective study [75] indicated that R2* quantification may serve as a substitute for measuring total body iron stores (determined by quantitative phlebotomies), suggesting that MRI can predict the number of phlebotomies needed for intensive therapy. These findings indicate that hepatic MRI R2* quantification, in addition to accurate measurement of the liver iron concentration, can also provide guidance for subsequent treatment in patients with hemochromatosis.

Iron overload can lead to significant morbidity and mortality if not appropriately managed. Therefore, accurate quantitative assessment of liver iron concentration plays a crucial role in risk stratification, guiding chelation therapy decisions, and monitoring treatment response in conditions such as hereditary hemochromatosis and transfusional iron overload. Clinical trials have provided preliminary evidence supporting the potential therapeutic use of thalidomide in treating thalassemia. Nevertheless, a research gap exists in the study of alterations in liver iron during thalidomide therapy. A previous study [76] sought to assess changes in liver iron content and volume in patients with transfusion-dependent ß-thalassemia receiving thalidomide treatment via R2* and SIR. These MRI methods could serve as noninvasive means to monitor liver iron levels. In this study, during the 12-month follow-up, patients exhibited a progressive increase in hemoglobin levels, erythrocyte counts, and platelet counts, as well as SIR_T1 and SIR_T2 values. Conversely, the serum ferritin levels, R2* values, and liver volume consistently decreased over the same period. The serum ferritin levels did not significantly decrease until the 12th month of thalidomide treatment. However, significant reductions in liver R2* values and significant increases in SIR_T1 and SIR_T2 values were observed as early as the 3rd month and continued through the 12th month of treatment. These findings suggest that thalidomide effectively alleviates whole-body iron overload over time and reduces liver iron deposition by the third month, preceding the decrease in serum ferritin levels. The study also revealed that the evaluation of MRI R2*, SIR_T1, and SIR_T2 may provide a reference for whether patients need to be treated with iron chelation during thalidomide treatment. These findings indicate that precise quantitative imaging techniques play crucial roles in evaluating the treatment response to iron overload in the liver and provide valuable guidance for subsequent therapeutic strategies.

The use of precise quantitative MR imaging techniques also plays a significant role in the prognosis of other diseases. A retrospective study [77] revealed that systemic iron overload (SIO), reflected by the LIC, is an independent negative prognostic factor for posttransplant outcomes in acute myeloid leukemia (AML) and myelodysplastic syndrome (MDS) patients undergoing allogeneic stem cell transplantation (allo-SCT). Therefore, MRI-based LIC and not interference-prone serum markers such as ferritin should be preferred for pretransplant risk stratification and patient selection in future clinical trials. Another retrospective cohort study [78] with the aim of evaluating iron overload in patients with sickle cell disease (SCD) and correlating SF with cardiac T2* MRI, liver T2* MRI, and LIC by R2-MRI revealed that the availability of oral chelation in parallel with the assessment of iron overload by MRI improved the management of iron overload in our population with SCD.

MRI-based techniques, particularly R2 and R2* measurements, have demonstrated their prognostic value in iron overload conditions. A higher liver iron concentration, as indicated by elevated R2 values, is associated with an increased risk of hepatic decompensation, hepatocellular carcinoma, cardiomyopathy, and mortality in patients with hemochromatosis. Furthermore, R2 and R2* can predict the response to phlebotomy or chelation therapy, with a higher baseline liver iron concentration being linked to a slower rate of iron removal. However, more direct comparisons between different hepatic iron quantification techniques and their impact on long-term clinical outcomes are still limited. Therefore, further prospective studies are necessary to validate these initial findings and fully integrate quantitative liver iron assessment into risk stratification and individualized therapy decisions for the optimal management of iron-related diseases.

In summary, quantitative hepatic iron assessment techniques, including MRI R2 relaxation and R2* measurements, hold promise for improving risk prediction, guiding precision therapy decisions, and monitoring treatment efficacy in patients with clinically significant iron overload. The incorporation of these quantitative assessments into clinical practice may help optimize care for patients affected by iron-related diseases.

## 4. Conclusions and Perspectives

Iron is an important microelement, and normal levels of iron are essential for maintaining homeostasis in the human body. However, the management of iron overload is challenging [79], largely because it often remains asymptomatic until substantial and irreversible end-organ damage occurs. Numerous studies have demonstrated a strong and linear correlation between LIC and systemic iron concentrations [80], offering a more practical approach to assess iron storage in the whole body. Compared with serological indicators with poor accuracy and invasive liver biopsy, CT- and MRI-based techniques have gained considerable attention in hepatic iron quantification because of their high sensitivity, noninvasiveness, and ease of follow-up for dynamic assessment. Nonetheless, it is undeniable that each technique has its limitations, and a comprehensive evaluation is essential for clinical application. Once a technique has been selected, consistency in the follow-up assessment method is vital to minimize systematic biases among different methodologies.

The emergence of new MRI-based sequences, such as UTE, QSM, Dixon, and CSE technologies, has significantly enhanced the potential of noninvasive liver iron quantification [72,81,82,83,84,85]. UTE sequences improve the accuracy of T2*/R2* methods across a broader range of LIC values [73,82], even under a high MR field. QSM stands out as the most sensitive technique for iron detection, with many applications in the brain, whereas it remains in the research stage for hepatic iron quantification due to respiratory motion. Moreover, the Dixon technique is able to provide a PDFF that effectively adjusts for fat interference in the MRI signal, ensuring reliable iron measurements in the presence of steatosis. Additionally, CSE technology, through its integration of complex chemical shift-encoded methods, offers advanced correction for noise, fat, and macroscopic B0 field inhomogeneities. This enables highly accurate and reproducible R2* measurements, making CSE a promising tool for precise LIC quantification in clinical practice.

Considering that R2-based FerriScan is currently the only FDA-approved method for LIC quantification [28,37], the establishment of internationally standardized imaging parameters, postprocessing procedures, and reporting protocols is urgent. Additionally, the development of new technologies will enhance the assessment and detection of other hemosiderin deposition diseases, such as hemophilic arthropathy [16]. These advancements facilitate the visualization of affected joints and the assessment of adjacent soft tissue involvement, owing to the superior soft tissue resolution of MR images. However, widespread clinical application still requires extensive validation, standardization, and adaptation of acquisition protocols to accommodate joint anatomy and mobility. Such standardization is crucial for facilitating data comparison and sharing across different studies, which in turn will lead to the formation of guidelines for the better management of patients with hepatic iron overload.

## Data Availability

No new data were created or analyzed in this study. Data sharing is not applicable to this article.

## Supplementary Materials

The following supporting information can be downloaded at: https://www.mdpi.com/article/10.3390/biomedicines12112456/s1, Table S1: The cutoff criteria for the severity of hepatic iron overload by several common methods; Table S2: Comparison of precise imaging quantification techniques for iron deposition in the liver. References [18,37,68,86,87] have been cited in the Supplementary Materials file.
