# Supplementary material for: Computed Tomography and Magnetic Resonance Imaging in Liver Iron Overload: From Precise Quantification to Prognosis Assessment
Source: Biomedicines. 2024 Oct 25;12(11):2456. doi: 10.3390/biomedicines12112456 (PMC11592092; doi:10.3390/biomedicines12112456)
Supplement: Supplementary file 1 [file biomedicines-12-02456-s001.zip › biomedicines-3141567-table s1.pdf]

Table S1. The cut-off criteria for severity of hepatic iron overload in several common methods.

	Methods	Normal	Abnormal			
			**Mild**	**Moderate**	**Severe**	**Extreme**
**Clinical methods**	SF (μg/L) [22]		>150 for menstruating females<br>>200 for males and non-menstruating females			
	TSAT (%) [23]		>45%			
	Liver biopsy (mg Fe/g dry tissue) [24]	<1.8	1.8–3.2	3.2–7.0	7.0–15.0	>15.0
**Imaging methods**	***CT***					
	Traditional CT (HU)		>70–75 HU			
	DECT-VIC (HU) [18]	<2.5	2.5–5.1	5.1–8.9	8.8–18.0	>18.0
	MRI					
	SIR	higher liver-to-muscle SIR	lower liver-to-muscle SIR			
	R2 at 1.5 T † (s^{-1}) [24]	45.2	45.2–63.5	63.5–102.1	102.1–161.3	>161.3
	R2 at 3.0 T * (s^{-1})	NA	NA	NA	NA	NA
	R2 * at 1.5 T ‡ (s^{-1}) [25]	75.3	75.3–129.1	129.1–275.1	275.1–582.4	>582.4
	R2 * at 3.0 T ‡ (s^{-1})	135.7	135.7–239.4	239.4–521.1	521.1–1114.2	>1114.2

Note: † Data were calculated according to the calibration curve from St Pierre TG et al. * FerriScan was the only commercially available R2 relaxometry technique for liver iron quantification at 1.5 T MRI, and no standardized calibration curve has been established for its application at 3.0 T MRI. ‡ Data were calculated according to the calibration curve from Hernando D et al. [25]. Abbreviations: SF = serum ferritin; TSAT = transferrin saturation; HU = Hounsfield units; DECT = dual-energy CT; VIC = virtual iron content; SIR = signal intensity ratio.
