# Supplementary material for: Computed Tomography and Magnetic Resonance Imaging in Liver Iron Overload: From Precise Quantification to Prognosis Assessment
Source: Biomedicines. 2024 Oct 25;12(11):2456. doi: 10.3390/biomedicines12112456 (PMC11592092; doi:10.3390/biomedicines12112456)
Supplement: Supplementary file 1 [file biomedicines-12-02456-s001.zip › biomedicines-3141567-table s2.pdf]

Table S2. Comparison of precise imaging quantification techniques for liver iron overload.

Techniques	Advantages	Limitations
Dual-Energy CT	Fast imaging speed<br>Relatively simple post-processing<br>The quantification of LIC regardless of the fat content<br>VIC value can be a potential index for accurately assessing liver iron accumulation	Insufficient quantitative accuracy<br>The potential risks associated with ionizing radiation
Liver-to-Muscle Signal Intensity Ratio (SIR)	The simplicity of implementation across multiple vendors and platforms<br>Widely available<br>Minimal postprocessing	The assumption that the reference tissue (paraspinal muscles) is normal<br>Not accurate for severe iron overload exceeding 350 μmol/g<br>Overestimate mild and moderate liver iron overload
T2 and R2 Relaxometry	Assessing a broad range of iron overload severities<br>Validated method with FerriScan	Availability solely at a 1.5 T field strength<br>A relatively long acquisition time (10–20 min)<br>Restricted spatial coverage of the liver<br>Susceptibility to motion artifacts
T2* and R2* Relaxometry	Wide availability with regulatory approval<br>Rapid acquisition time<br>Well validated, reproducible<br>Highly linear relationship between R2* and LIC<br>Application at a 1.5 T and 3.0 T field strength	Vulnerability to various confounding factors<br>Not well-suited for quantification of LIC greater than approximately 40 mg/g (700 μmol/g) at 1.5 T or approximately 26 mg/g (466 μmol/g) at 3.0 T

Note: LIC = liver iron concentration; VIC = virtual iron content.
